# Pneumomediastinum Complicating Diabetic Ketoacidosis and Boerhaave's Syndrome

**DOI:** 10.1155/2013/598720

**Published:** 2013-11-13

**Authors:** Samer Alkhuja, Natalya Gazizov, Gervais Charles

**Affiliations:** The Commonwealth Medical College, Pocono Medical Center, 175 East Brown Street, Suite 203, East Stroudsburg, PA 18301, USA

## Abstract

An 18-year-old man presented with altered mental status. He was found to have diabetic ketoacidosis. Chest X-ray showed pneumomediastinum. After intubation for air-way protection, an oral-gastric tube was placed. A chest computed tomography scan showed the tip of the oral-gastric tube to be in the right hemithorax. The patient underwent a thoracotomy and was managed in the intensive care unit. Both diabetic ketoacidosis and Boerhaave's syndrome should be considered as possible causes of pneumomediastinum in a patient with similar presentation. Boerhaave's syndrome should be ruled out prior to the insertion of an oral-gastric tube to avoid further morbidities.

## 1. Case

An 18-year-old man was brought to the emergency department (ED) because of altered mental status. Family reported that he had nausea and vomiting before presenting to the ED. Past medical history was significant for diabetes, diabetic ketoacidosis (DKA), and hypothyroidism. Past surgical and social histories were unremarkable. Home medication was insulin glargine.

Physical exam revealed a lethargic patient. Vitals were blood pressure 130/70 mmHg, pulse 96/minute, respiration 28/minute, temperature was normal. Lung auscultation showed left basal crackles. 

Chest radiograph (CXR) showed lower lobe infiltrates and pneumomediastinum (PM). Laboratory results: Arterial blood gas: PH 6.930 (7.350–7.450), PCO_2_ 16 mmHg (35–45), PO_2_ 138 mmHg (81–110), and serum bicarbonate 3 mmol/L (22–26); serum electrolytes: sodium 129 mmol/L (136–144), potassium 6.4 mmol/L (3.3–5.1), and chloride 100 mmol/L (101–111); glucose 1087 mg/dL (60–250), blood urea nitrogen 40 mg/dL (8–26), and creatine 2.68 mg/dL (0.70–1.20). Blood counts were normal.

The patient was intubated for airway protection because of altered mental status. A postintubation CXR showed PM, lower lobe infiltrates, and an air-filled distended stomach. An oral-gastric (OG) tube was placed easily, to decompress the air-filled distended stomach after bagging prior to intubation and in view of nausea and vomiting. A chest computed tomography scan showed multilobar infiltrates suggestive of aspiration pneumonia, PM, and the tip of the OG tube to be in the right hemithorax. The patient underwent a right thoracotomy with the intention to repair the esophageal rupture. Intraoperatively, the tip of the OG tube was found to be retracted into the esophageal lumen. Inspection of the esophagus did not reveal a clear rupture. An OG tube was placed intraoperatively, and a chest tube was placed into the right pleural space.

The patient was given intravenous (IV) fluid for hydration, IV insulin drip, IV metronidazole, and IV levofloxacin. Serial CXRs showed clearing of pneumonic infiltrates and PM. The chest tube was discontinued, and the patient was successfully extubated. After extubation, an esophagogram was performed and showed no extravasation of contrast.

## 2. Discussion

PM is a rare comorbidity of DKA. Other reported pneumo-complications in patients with DKA are pneumothorax [1], pneumopericardium, and pneumorrhachis (epidural pneumatosis) [2–4]. Pneumorrhachis may result from gas passing from the posterior mediastinum through the intervertebral foramina into the epidural space [2]. PM is characterized by the presence of air in the mediastinum, secondary to alveolar rupture that allows air to traverse along the bronchovascular bundle which may lead to PM or pneumopericardium [2–5]. Acidotic (Kussmaul) breathing alone may apparently induce transalveolar pressure swings that are sufficient to cause alveolar rupture [2–5]. Sever vomiting may result in esophageal rupture and the development of PM [5, 6]. If air collections accumulate in the pharynx and larynx, the voice may change (“hot potato voice”). Hamman's sign may be present [7]. Most of the clinical signs of PM or pneumopericardium, are abscent after correcting the DKA [2]. 

Pauw et al. conducted an extensive review of the literature and found that esophageal rupture (Boerhaave's syndrome) was not detected in any of the reported cases and suggested that the mechanism of PM is alveolar rupture [2]. The PM in our patient was initially thought to be due only to alveolar rupture in the process of DKA. Although an iatrogenic esophageal perforation with the OG tube cannot be fully excluded, the OG tube was placed easily making an iatrogenic cause less likely. The OG tube was displaced through the esophagus into the right hemithorax, which raises the possibility of Boerhaave's syndrome secondary to nausea and vomiting which the patient experienced prior to presentation. 

## 3. Conclusion

This case illustrates the need to consider both of DKA and Boerhaave's syndrome as possible causes of PM in a patient who has a similar presentation. If the clinical scenario necessitates placement of an OG tube, Boerhaave's syndrome should be excluded prior to the insertion, to avoid displacing the OG tube through the esophageal wall into the pleural space. Esophageal gastroendoscopy is only indicated when other pathologies are being considered and after healing of the rupture is documented with a water-soluble contrast esophageal swallow [2]. 
